# Video-Assisted Thoracoscopy is Superior to Standard Computer Tomography of the Thorax for Selection of Patients With Spontaneous Pneumothorax for Bullectomy

**DOI:** 10.1155/DTE.2.89

**Published:** 1995

**Authors:** Julius P. Janssen, Richard P. Golding, Miguel A. Cuesta, Thomas Sutedja, Pieter E. Postmus

**Affiliations:** 1 Department of pulmonology Free University Hospital De Boelelaan 1117 Amsterdam The Netherlands; 2 Department of Radiology Free University Hospital Amsterdam The Netherlands; 3 Department of Surgery Free University Hospital Amsterdam The Netherlands

## Abstract

*Background*: Spontaneous pneumothorax (SP) is a common disease of unknown cause often attributed to rupture of a subpleural bulla or bleb [in this study described as emphysema-like changes (ELC)]. Treatment of SP varies from conservative (rest) to aggressive (surgery). Patients with bullae >2 cm diameter, found either by chest roentgenogram or during thoracoscopy, are often treated surgically (bullectomy and pleurectomy, or abrasion). Thoracoscopy is frequently used as the method of choice to select patients for surgery. With the recent introduction of video-assisted thoracoscopy (VAT), it is now possible to combine a diagnostic and therapeutic procedure. However, to do this general anesthesia and a fully equipped operating theater are needed. Proper selection of patients for this costly and time-consuming procedure is necessary. We evaluated whether standard computed tomography (CT) is appropriate for selection of patients with SP who are candidates for surgical intervention.

*Methods*: In 43 patients with SP, CT was performed after re-expanding the lung by suction through a chest tube if the lung was completely collapsed. After <48 hours VAT under general anesthesia was performed. All CT scans were scored by two investigators who were not informed about the VAT findings or the outcome of the patient. CT findings and VAT findings were compared.

*Results*: In 16 patients (37%), CT scans of the affected lung were considered normal, while in 13 patients (30%) ELCs ≥2 cm were seen and in 14 patients (33%) ELCs <2 cm were found. VAT showed a normal lung in 11 patients (26%), in 24 patients ELCs ≥2 cm were seen, and in 8 patients ELCs <2 cm were present. Of these 32 patients, in 18 bullous degeneration of the apex of the upper lobe was found. Of the 24 patients with ELCs ≥2 cm detected during VAT, 13 were detected by CT. In no patient were ELCs ≥2 cm seen on CT scans that were not detected during VAT. The sensitivity of CT for ELCs ≥2 cm is 54%, and the specificity is 100%. The positive predictive value is 100%; the negative predictive value is 63%.

*Conclusions*: CT detects ELCs ≥2 cm in 54% of the patients in whom VAT shows these abnormalities. If interventional therapy is deemed appropriate for first time or recurrent SP, VAT should be used since it is superior to CT in demonstrating the presence, number, and size of ELCs.


*Diagnostic and Therapeutic Endoscopy*, 1995, Vol. 2, pp. 89–92
Reprints available directly from the publisher
Photocopying permitted by license only

© 1995 Harwood Academic Publishers GmbH
Printed in Singapore

### Video-Assisted Thoracoscopy Is Superior to Standard Computer Tomography of the Thorax for Selection of Patients with Spontaneous Pneumothorax for Bullectomy

JULIUS P. JANSSEN, RICHARD P. GOLDING, MIGUEL A. CUESTA, THOMAS SUTEDJA and PIETER E. POSTMUS

*Departments of pulmonology (J.P.J., T.S., P.E.P.), Radiology (R.P.G.), and Surgery (M.A.C.), Free University Hospital, Amsterdam, The Netherlands*

*(Received March 3, 1995; in final form April 4, 1995)*

*Background*: Spontaneous pneumothorax (SP) is a common disease of unknown cause often attributed to rupture of a subpleural bulla or bleb [in this study described as emphysema-like changes (ELC)]. Treatment of SP varies from conservative (rest) to aggressive (surgery). Patients with bullae >2 cm diameter, found either by chest roentgenogram or during thoracoscopy, are often treated surgically (bullectomy and pleurectomy, or abrasion). Thoracoscopy is frequently used as the method of choice to select patients for surgery. With the recent introduction of video-assisted thoracoscopy (VAT), it is now possible to combine a diagnostic and therapeutic procedure. However, to do this general anesthesia and a fully equipped operating theater are needed. Proper selection of patients for this costly and time-consuming procedure is necessary. We evaluated whether standard computed tomography (CT) is appropriate for selection of patients with SP who are candidates for surgical intervention.

*Methods*: In 43 patients with SP, CT was performed after re-expanding the lung by suction through a chest tube if the lung was completely collapsed. After <48 hours VAT under general anesthesia was performed. All CT scans were scored by two investigators who were not informed about the VAT findings or the outcome of the patient. CT findings and VAT findings were compared.

*Results*: In 16 patients (37%), CT scans of the affected lung were considered normal, while in 13 patients (30%) ELCs ≥2 cm were seen and in 14 patients (33%) ELCs <2 cm were found. VAT showed a normal lung in 11 patients (26%), in 24 patients ELCs ≥2 cm were seen, and in 8 patients ELCs <2 cm were present. Of these 32 patients, in 18 bullous degeneration of the apex of the upper lobe was found. Of the 24 patients with ELCs ≥2 cm detected during VAT, 13 were detected by CT. In no patient were ELCs ≥2 cm seen on CT scans that were not detected during VAT. The sensitivity of CT for ELCs ≥2 cm is 54%, and the specificity is 100%. The positive predictive value is 100%; the negative predictive value is 63%.

*Conclusions*: CT detects ELCs ≥2 cm in 54% of the patients in whom VAT shows these abnormalities. If interventional therapy is deemed appropriate for first time or recurrent SP, VAT should be used since it is superior to CT in demonstrating the presence, number, and size of ELCs.

**KEY WORDS:** Computed tomography, pneumothorax, video-assisted thoracoscopy

### INTRODUCTION

Spontaneous pneumothorax (SP) is a relatively common disease of unknown cause. In more than half of the patients who underwent thoracoscopy or thoracotomy, pathologic changes, mostly subpleural blebs or bullae, are found (1–5), which might be related to the cause of SP.[6] Why sudden rupture of a bleb or bulla occurs is unknown. In the literature, the terms "blebs" and "bullae" are often confusingly interchanged. Despite their proper anatomic definition, in some studies, blebs are defined as lesions with a diameter smaller than 2 cm, and bullae as lesions larger than 2 cm (7). Recently Bense and co-workers (8) introduced the term "emphysema-like changes" (ELC), which causes less confusion and is therefore to be preferred.

Treatment of SP varies from conservative to invasive with some advocating bedrest, others drainage, and yet

Address for correspondence: Pieter E. Postmus M.D. Ph.D., F.C.C.P., Department of Pulmonology, Free University Hospital, De Boelelaan 1117, Amsterdam, The Netherlands.

others bullectomy and pleurectomy at thoracotomy. The role of surgery especially in patients with SP is not clearly defined. In a number of studies thoracoscopy has been performed to select patients who might benefit from surgical intervention. In particular, patients with ELCs larger than 2 cm are considered good candidates for bullectomy, either by lateral or axillary thoracotomy (3,9).

Recently, video-assisted thoracoscopic (VAT) surgery has been introduced as a less invasive alternative to thoracotomy (10). Selection of suitable patients for bullectomy is not possible with standard chest roentgenograms, due to their low sensitivity (7). Although computed tomography (CT) of the thorax has been proven to be much more sensitive in the diagnosis of ELCs than conventional chest roentgenograms, its correlation with thoracoscopic findings in patients with SP has so far not been prospectively studied. In this report, we compare the results of CT with the results of VAT in patients with SP with special emphasis on the potential value of CT for selection of patients for bullectomy.

### MATERIALS AND METHODS

Forty-three patients admitted for SP without known underlying pulmonary disease were studied. If the lung was collapsed, re-expansion was attempted by chest tube drainage. CT scanning (Somatom Plus, Siemens, Erlangen, Germany) was performed with the patient supine in full inspiration with 10-mm contiguous slices with 255 mA and 120 kV and reconstructed on a 512 × 512 matrix. All patients gave informed consent.

VAT was performed under general anesthesia with selective intubation within 2 days after CT. The pleural cavity and the lung were thoroughly inspected. If a normal visceral pleura or ELCs <2 cm were found, talc pleurodesis and electrocoagulation of the ELCs was performed. ELCs ≥2 cm were removed by thoracoscopic bullectomy and scarification of the parietal pleura was done by electrocoagulation at the level of the first five ribs. Afterwards the CT scans were independently reviewed by two experienced observers (R.P.G. and P.E.P.), without the knowledge of the results of VAT. The CT findings were compared with the results of VAT, the "golden standard."

### RESULTS

### CT Findings

In five patients CT scanning was performed while the lung was not completely expanded. In 16 patients (37%), CT scans of the affected lung were considered normal, while in 13 patients (30%) ELCs ≥2 cm were seen and in 14 patients (33%) ELCs <2 cm were found.

In nine patients (21%), ELCs were detected under the level of the carina. All of these patients also had ELCs in the upper half of the lung. In 14 patients (33%), ELCs were visible in both lungs. In one patient, ELCs were seen only in the unaffected lung.

### VAT Findings

ELCs <2 cm were found in 8 patients and ELCs ≥2 cm in 24 patients (Fig. 1). No abnormalities were seen in 11 patients. In 18 of the patients with ELCs, bullous degeneration of the apex of the upper lobe was found.

### Comparison of CT and VAT (Table 1)

*ELCs ≥2 cm*

Of the 24 patients with ECLs detected during VAT, in 13 these were detected by CT. All lesions found by CT were also found during VAT. The sensitivity of CT is 54% and the specificity is 100%. The positive predictive value (PV+) is 100%; the negative predictive value (PV–) is 63%.

*All ELCs*

In 27 patients the CT scan demonstrated these changes adjacent to the visceral pleura; in 24 of these patients this was confirmed during VAT. However, in three patients no changes were found during VAT, and the CT findings in these patients were considered as false-positive. In one of these three patients, CT was done while the lung was still partly collapsed. In eight patients with a normal CT scan, ELCs were found during VAT; these CT scans were scored as false-negative. In four of these patients a bullous degeneration of the apex was found, in two patients multiple small ELCs were found, and in two patients one small ELC was detected. In three patients both CT and VAT findings were normal. This results in a sensitivity of 75% and a specificity of 73% for CT to find ELCs adjacent to the pleura. The PV+ is 88%, and the PV– is 50%.

With regard to the number of ELCs in a patient, CT matched VAT completely in 22 (51%) cases. CT underestimated the number of ELCs in 16 cases (37%); in one of these patients CT was performed while the lung was still partly collapsed. In five cases (11%) more ELCs were found on CT than during VAT.

### DISCUSSION

Conventional thoracoscopy, without the advantages of modern equipment and video monitoring, showed pathologic changes in 60 to 70% of all patients with SP (11).

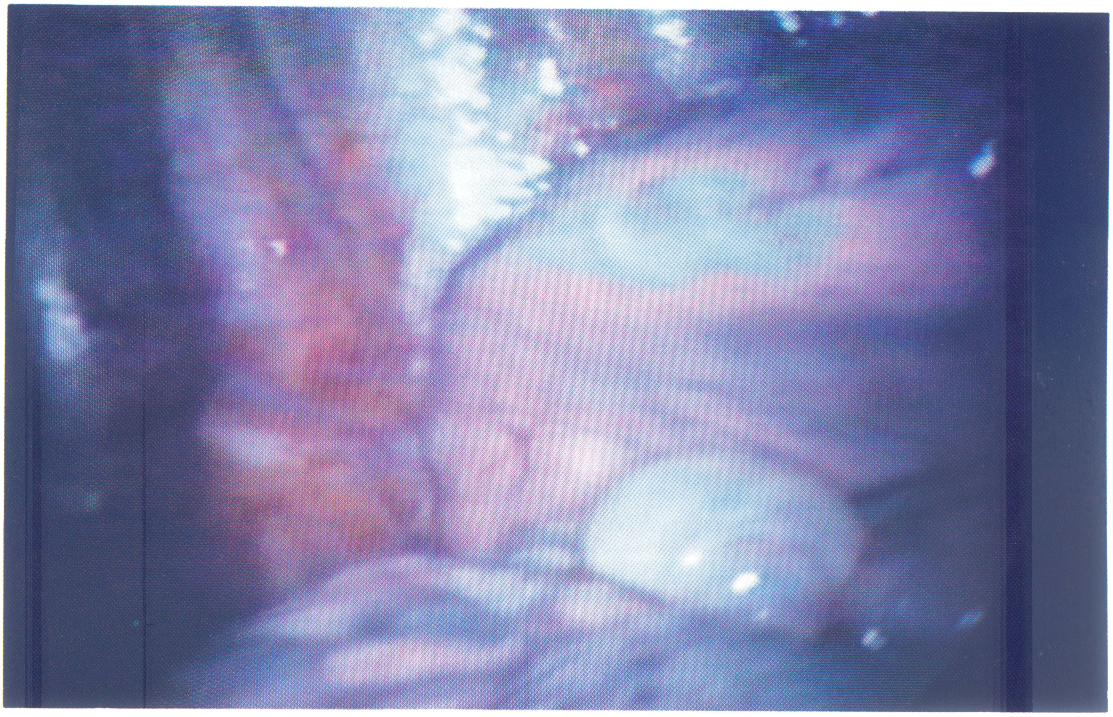

**Figure 1** Endoscopic view of the lung with small ELCs.

**Table 1** Results of VAT and CT

| | CT | | | |
|---|---|---|---|---|
| *VAT* | *Normal* | *ELCs <2* | *ELCs >2* | *Total* |
| VAT | | | | |
| Normal | 8 | | | 11 |
| ELCs <2 | 4 | | | 8 |
| ELCs ≥2 | 4 | | 13 | 24 |
| Total | 16 | | 13 | 43 |

With CT in a number of studies, ELCs were found in patients treated for SP. The incidence of these abnormalities was higher in patients treated surgically for SP and with recurrent SP than in those with uncomplicated SP (12,13). The frequency of these ELCs was >80% in a small group of patients with idiopathic SP (8). CT appears to be somewhat more sensitive than conventional thoracoscopy in the diagnosis of ELCs in patients treated for SP. In our study VAT was used instead of conventional thoracoscopy. We found VAT to be more accurate than CT in the diagnosis of ELCs at the time of SP. In 32 (74%) patients, ELCs were found during VAT versus 27 (63%) using CT scanning. Of these 27, only 24 were confirmed by VAT. CT underestimated the number of ELCs in 37% ($n$ = 16) of the patients with abnormalities detected during VAT. However, in five cases more lesions were found on CT than found during thoracoscopy. It is not clear if this was due to false-positive CT scans, or false-negative thoracoscopic findings.

Some observations may explain why more ELCs were seen during VAT. First, most ELCs are expected in the apical part. This is often collapsed in patients seen with SP and can be missed on CT scanning of a partially collapsed lung. Secondly, small changes can be missed on the CT scan, when using 10-mm slices as in this study. Thirdly, inflation and deflation of the lung during VAT may help in demonstrating the number and size of ELCs. Lastly, a torn bulla will not re-expand after chest tube drainage and can be missed on the CT scan.

Can CT scanning replace VAT for the selection of patients with SP for surgical intervention? Currently this would not seem to be the case. In our study only a little more than half of the patients with lesions >2 cm were detected by CT scans. With some improvements in the scanning technique, it might be possible to improve this result. The use of thinner slices would improve the sensitivity of the CT scan but increases the radiation dose. A spiral CT scan during a Valsalva maneuver is another refinement. ELCs in the lower lobes are always found together with abnormalities in the upper lobes; therefore, for SP patients, scanning of the upper half of the thorax will be sufficient.

We conclude that if interventional therapy is deemed appropriate for first time or recurrent SP, VAT should be used since this is superior to CT in demonstrating the presence, number, and size of ELCs.

### REFERENCES

1. Janssen JP, van Mourik J, Cuesta Valentin M, *et al.* Treatment of patients with spontaneous pneumothorax during videothoracoscopy. Eur Resp J 1994;7:1281–1284.
2. Swierenga J, Wagenaar JPM, Bergstein PGM. The value of thoracoscopy in the diagnosis and treatment of diseases affecting the pleura and lung. Pneumologie 1974;151:11–18.
3. Boutin C, Viallat JR, Aelony Y. Practical thoracoscopy. Berlin: Springer Verlag, 1991:73–81.
4. Vanderschueren RGJRA. Le talcage pleural dans le pneumothorax spontané. Poumon Coeur 1981;37:273–276.
5. Baronofsky IF, Warden HG, Kaufman J, *et al.* Bilateral therapy for unilateral spontaneous pneumothorax. J Thorac Cardiovasc Surg 1957;34:310–322.
6. Getz SB, Beasley WE. Spontaneous pneumothorax. Am J Surg 1983;145:823–827.
7. Mithlehner W, Friedrich M, Dissmann W. Value of computer tomography in the detection of bullae and blebs in patients with primary spontaneous pneumothorax. Respiration 1992;59: 221–227.
8. Bense L, Lewander R, Eklungd G, *et al.* Nonsmoking, non-alpha$_1$-antitirypsin deficiency-induced emphysema in nonsmokers with healed spontaneous pneumothorax, identified by computed tomography of the lungs. Chest 1993;103:43–48.
9. Olsen PS, Andersen HO. Long-term results after tetracycline pleurodesis in spontaneous pneumothorax. Ann Thorac Surg 1992;53:1015–1017.
10. Bagnato VJ. Treatment of recurrent spontaneous pneumothorax. Surg Laparosc Endosc 1992;2:100–103.
11. Brekel van de JA, Duurkens VAM. Vanderschueren RGJRA. Pneumothorax. Results of thoracoscopy and pleurodesis with talc poudrage and thoracotomy. Chest 1993;103:345–347.
12. Warner BW, Bailey WW, Shipley RT. Value of computed tomography of the lung in the management of primary spontaneous pneumothorax. Am J Surg 1991;162:39–42.
13. Lesur O, Delorme N, Fromaget CM, Bernadac P, Polu JM. Computed tomography in the etiologic assessment of idiopathic spontaneous pneumothorax. Chest 1990;98:341–347.

